# Microglial deletion of C1q reduces phagocytosis of amyloid and synapses in an AD mouse model

**DOI:** 10.1002/alz.086248

**Published:** 2025-01-03

**Authors:** Tiffany J Petrisko, Shu‐Hui Chu, Andrea J Tenner

**Affiliations:** ^1^ University of California, Irvine, Irvine, CA USA

## Abstract

**Background:**

The complement system contributes to enhanced inflammation and cognitive decline in Alzheimer’s disease (AD). Previous studies have demonstrated constitutive deletion of the classical initiator protein, C1q, reduces glial activity and attenuates neuronal loss in AD mouse models. As it is now known that microglia are the primary producers of C1q in the brain, the objective of this study was to determine if microglial specific deletion of C1q would reduce lysosome associated phagocytosis of Vglut1, an excitatory synapse marker, and if reductions in the phagocytosis of Vglut1 would be accompanied by a reduction in the phagocytosis of beta‐amyloid.

**Method:**

Briefly, C1qa^FL/FL^CX3CR1Cre (designated C1q^ΔMG^) mice, which show deletion of microglial C1q by 2 months of age, were crossed to ArcticC1qa^FL/FL^ mice to generate WT and Arctic (Arc) C1qa^FL/FL^ mice with and without the CX3CR1Cre transgene. Brains were then collected at 10m of age to study amyloid phagocytosis by microglia and astrocytes as well as microglial phagocytosis of Vglut1 using immunofluorescence. Briefly, sections were co‐labeled for microglia (Iba1) or astrocytes (GFAP), lysosomes (CD68 or Lamp2), and amyloid (6E10) or Vglut1. Confocal z‐stacks of amyloid plaques throughout the hippocampus were acquired at 40X using a 3.0 Zoom while microglia were imaged at 63X with a 3.5 Zoom in the CA1 subregion of the hippocampus.

**Results:**

Microglial engulfment (Iba1/6E10 co‐localization) and lysosome associated phagocytosis (Iba1/6E10/CD68) was significantly impaired in Arctic mice lacking C1q (p<0.05). While astrocytes did not change their engulfment of amyloid (GFAP/6E10 colocalization), lysosome associated phagocytosis of amyloid was significantly impaired in Arc C1q^ΔMG^ astrocytes. Arctic mice displayed increased phagocytosis of Vglut1 by microglia as expected, and the deletion of C1q from microglia significantly decreased this engulfment. Unexpectedly, WT C1q^ΔMG^ displayed increased Vglut1 phagocytosis compared to WT controls. No changes were observed in the percentage of Vglut1 puncta.

**Conclusion:**

Loss of microglial C1q reduces amyloid uptake by astrocyte and microglia and in the CA1 region, lack of microglial C1q also reduces Vglut1 association with the lysosomes in the Arctic AD mouse model.

